# Frailty and Comorbidities Among Young Adult Cancer Survivors Enrolled in an mHealth Physical Activity Intervention Trial

**DOI:** 10.21203/rs.3.rs-3111745/v1

**Published:** 2023-06-28

**Authors:** Erin M Coffman, Andrew B. Smitherman, Erik A. Willis, Dianne S. Ward, Deborah F. Tate, Carmina G Valle

**Affiliations:** University of North Carolina at Chapel Hill

**Keywords:** young adult, cancer survivors, frailty, comorbidities

## Abstract

**Purpose:**

The physical frailty phenotype identifies individuals at risk for adverse health outcomes but has rarely been assessed among young adult cancer survivors (YACS). This study describes frailty status among YACS participating in a PA intervention trial.

**Methods:**

YACS were categorized by frailty status at baseline using the 5-item FRAIL index: fatigue; weight loss; illness; ambulation; resistance. Chi-square tests compared frailty and comorbidities by characteristics. Prevalence rates (PRs) for the independent associations between characteristics, frailty, and comorbidities were estimated using modified Poisson regression models.

**Results:**

Among 280 YACS (82% female, M=33.4±4.8 years, M=3.7±2.4 years post-diagnosis), 14% had frailty, and 24% prefrailty; the most frequent criteria were fatigue (70%), resistance (38%), and ambulation (14%). Compared to BMI <25, higher BMI (BMI 25–30, PR: 1.65, 95% CI: 1.02–2.65; BMI > 30, PR: 2.36, 95% CI: 1.46–3.81) was associated with increased frailty status. Compared to 0, 1–50 minutes/week of moderate-to-vigorous PA was associated with reduced frailty (PR: 0.62, 95% CI: 0.43–0.90). Most YACS (55%) reported ≥ 1 comorbidity, most frequently depression (38%), thyroid condition (19%), and hypertension (10%). Men were less likely to report comorbidities (PR: 0.63, 95% CI: 0.42–0.93). Current/former smokers (PR: 1.29, 95% CI: 1.01–1.64) were more likely to have comorbidities.

**Conclusion:**

Prevalence of frailty and comorbidities in this sample was similar to other YACS cohorts and may be an indicator of accelerated aging and increased risk for poor outcomes.

**Implications for Cancer Survivors:**

Assessment of frailty may help identify YACS at risk for adverse health outcomes.

## INTRODUCTION

Each year in the United States an estimated 80,00 young adults (diagnosed ages 20–39) are diagnosed with cancer.[1] Due to advances in prevention, screening and treatment, the overall 5-year relative survival rate for those diagnosed in this age group is 85%, with variation across cancer types.[2] However, these relatively young cancer survivors are at increased risk for late effects (i.e., organ damage or treatment-related conditions emerging months or years after treatment), including chronic health conditions, secondary cancers, and premature mortality.[3] Compared to age- and sex-matched controls without a history of cancer, young adult cancer survivors (YACS) have a 2–3 fold increased risk for cardiomyopathy, stroke, premature ovarian failure, chronic liver disease, and renal failure.[4] By ten years post treatment, over 40% of YACS have ≥ two comorbidities compared to 20% for age-matched peers without a history of cancer.[4] These comorbidities may be indicators of premature aging or frailty.

Frailty, generally considered a condition of aging, is a medical syndrome characterized by diminished physiological capacity that increases an individual’s susceptibility to physiological stressors, resulting in loss of independence, and death.[5] The physical frailty phenotype is commonly assessed using five criteria: fatigue, weakness, slowness, low energy expenditure, and unintentional weight loss.[6] Frailty status is classified based on number of criteria present—zero and one as non-frail, two as prefrail, and more than two as frail.[7] The frailty phenotype identifies those who are at risk for chronic diseases, adverse health outcomes, and is a predictor of premature mortality.[7], [8] Most evidence to date demonstrates that frailty is associated with age-related functional decline in older populations, yet it has not been studied extensively in the YACS population.

Ness et al. first assessed the prevalence of frailty among adult survivors of childhood cancer (n = 1,922; mean age 33.6 SD 8.1 years; diagnosed < 21 years old, survived ≥ 10 years) in the St. Jude Childhood Cancer Survivorship Cohort (CCSC) in 2013. The prevalence of prefrailty and frailty among this retrospective cohort were 31.5% and 13.1% among women and 12.9% and 2.7% among men, respectively. [9] This is comparable to the prevalence of frailty among individuals over the age of 65 without a history of cancer of 9.9%.[9] Smitherman et. al first characterized the Fried physical frailty phenotype of AYA cancer survivors (15–39 years old at diagnosis, at least 1 year post-diagnosis) using the University of North Carolina (UNC) Cancer Survivorship Cohort and found 10% met the criteria for frailty and 21% for prefrailty.[10] In both of these cohorts, the prevalence of prefrailty/frailty was associated with age, sex, number of comorbid conditions, and body mass index, with associations similar to those found in noncancer and cancer cohorts of adults over the age of 60.[9], [10] Obesity, a number of comorbidities (i.e., high cholesterol, hypertension, arthritis, diabetes), and anxiety or depression are all positively associated with increased frailty risk among older adults.[7], [8] These factors are highly prevalent among YACS, but little is known about whether these factors are associated with increased risk of frailty in YACS. [9], [10] Understanding the prevalence of frailty and underlying risk factors for frailty among this population may help to inform effective interventions to slow or prevent functional decline and adverse health outcomes.

Thus, the goal of this study is to characterize frailty status and non-cancer comorbidities in a sample of YACS and to identify baseline characteristics associated with frailty status and comorbidities.[9]–[11] We conducted a secondary analysis of data from the Improving Physical Activity after Cancer Treatment (IMPACT) trial, a randomized controlled trial of a mobile physical activity intervention among YACS using an established frailty measurement tool, the FRAIL survey-based index to assess physical frailty phenotype.

## METHODS

### Participants and Data

Data are from YACS enrolled in the IMPACT trial who completed baseline assessments and participated in a 12-month randomized controlled trial. The IMPACT trial, described elsewhere,[12] evaluated a theory-based mobile physical activity intervention with adaptive goal-setting and tailored feedback using digital tools, compared to a self-help control group, among 280 YACS. Participants were recruited, screened, and randomized between August 2018 and October 2019. Potentially eligible individuals were recruited from around the United States using a variety of methods including social media advertisements, direct mailings, community-based partnerships, and clinics. [13] Inclusion criteria included diagnosis with invasive malignancy between the ages of 18–39, diagnosis within the last 10 years with no evidence of progressive disease or secondary primary cancers, and not adhering to guideline recommendations of 150 minutes/week of moderate-to-vigorous physical activity. Participants were excluded if they had a history of heart attack or stroke within the past 6 months, psychiatric diagnosis requiring hospitalization in the past year, past diagnosis or treatment of an eating disorder, or health problems that would preclude ability to walk for physical activity. Untreated hypertension, hyperlipidemia, and diabetes required permission of their health care provider. Additional inclusion and exclusion criteria are reported elsewhere. [12] Participants were assessed prior to randomization. Study procedures were reviewed and approved by the Protocol Review Committee of the UNC Lineberger Comprehensive Cancer and the Institutional Review Board at The University of North Carolina at Chapel Hill (Study #: 16-3409).

### Data Collection and Measures

After each participant consented to participate, study staff emailed individuals a unique link to baseline questionnaires using REDCap (Research Electronic Data Capture), a secure, web-based software platform.[14], [15] Baseline questionnaires included participant demographics, health history including cancer type, stage, time since diagnosis, treatment type(s), medications, and lifestyle.

### Outcomes

The prevalence of the prefrailty and frailty were of primary interest for this analysis. Frailty was assessed using the FRAIL index, a survey-based measurement of the physical frailty phenotype that evaluates five components using items from commonly collected patient reported outcome (PRO) measures and has demonstrated sensitivity for identifying individuals with physical deficits (i.e., Fatigue, Resistance, Ambulation, Illnesses, and Loss of Weight) that correlate to the five components of the physical frailty phenotype (i.e., fatigue, weakness, slowness, low energy expenditure, and unintentional weight loss).[5], [16] The index was originally created as a means to identify frailty without requiring in-person assessments, making repeat measurements and evaluations of large samples feasible.[16] Index items were chosen that demonstrated significant correlations with markers classically associated with frailty (i.e., grip strength, gait speed, etc.) and has demonstrated strong convergent and predictive validity in cross-sectional analyses.[16] The FRAIL Index measure used in our study is consistent with that originally described, using PRO measures available.[10] Index items were from the Medical Outcomes Study 36-Item Short Form (SF-36)), a 36-item survey that assesses health-related quality of life across eight health concepts: physical function, pain, role limitations due to physical or emotional health, emotional well-being, social functioning, energy/fatigue, and general health perceptions.[17] The number of positive responses for each of five items described below were summed to create the FRAIL index value (range 0–5). For each item, positive responses were scored as 1-present, or 0-absent. A value of ≥ 3 was considered frail, a value of 2 was considered pre-frail, and values < 2 were considered non-frail.

*Fatigue* was evaluated using the 4-item vitality subscale of the Medical Outcomes Survey Short Form–36 (SF-36), relating to energy/fatigue (e.g., “How much of the time during the past 4 weeks...did you feel full of pep?”) with 6-point Likert responses ranging from none of the time to all of the time. These items were averaged and scores 1.3 standard deviations below the US adult population mean of 52.15 were coded as 1 indicating fatigue was present, while scores above this cut point were coded as 0.[17]

*Resistance* was assessed with the SF-36 question, “Climbing several flights of stairs, does your health now limit you a lot, limit you a little, or not limit you at all?” Responses of “yes, a lot” or “yes, a little” indicated difficulty and were coded as 1, while response of “No, not limited at all” was coded as 0.[17]

*Ambulation* was measured with the SF-36 question, “Walking several blocks does your health now limit you a lot, limit you a little, or not limit you at all? Responses of “yes, a lot” or “yes, a little” indicated difficulty with ambulation and were coded as 1, while response of “No, not limited at all” was coded as 0. [17]

#### Illnesses (e.g., comorbidities).

Self-reported comorbidities were assessed by health history questionnaires. Participants were asked “Have you ever been told by a doctor or other health care professional that you have any of the following conditions: high blood pressure, high cholesterol, type 1 diabetes mellitus, type 2 diabetes mellitus, thyroid disorder, heart disease, stroke, heart attack, cancer, depression, other psychological disorder, or eating disorder.” Responses were summed with ≥ 5 positive responses indicating coded as 1, and < 5 comorbidities were coded as 0.

#### *Weight Loss* > 5%.

Two questions about health history (“What is your current weight (in pounds)?” and “What was your weight 6 months ago?”) were used determine if weight change in the last 6 months indicated a clinically significant amount of weight loss.[18] Percent change in weight was calculated by subtracting current weight from weight 6 months ago divided by weight 6 months ago. Self-reported weight loss of ≥5% was coded as 1, and < 5% coded as 0.

### Other Variables

Baseline demographic, health, and lifestyle characteristics that have previously been shown to be associated with frailty status were chosen to describe the sample and serve as independent variables in multivariate models.[8], [11], [19]

#### Demographics.

Basic demographic information including age, sex, race, ethnicity, education level, employment status and income were self-reported at baseline.

#### Anthropometric measures.

Height was self-reported at baseline using a single item from the Behavioral Risk Factor Surveillance System survey[20] adapted for use in the NCI’s Health Information and National Trends Survey[21]. Self-reported height has shown to be highly correlated with measured height in young adults.[22] Height and self-reported weight were used to calculate *body mass index* (kg/m^2^).

#### Baseline physical activity.

Self-reported physical activity was assessed using a modified version of the leisure score index of the Godin Leisure Time Exercise Questionnaire which has demonstrated reliability and validity when compared to several other self-report exercise measures and objective measures in different populations.[23], [24] Participants report frequency of light, moderate and vigorous exercise, and average duration (minutes) during a typical week. Self-reported moderate and vigorous exercise were combined for minutes of moderate-to-vigorous physical activity (MVPA) per week.

#### Medical History.

Participants answered questions regarding their cancer history, including cancer type, cancer stage, time since diagnosis, and treatment type. In addition to the previously described comorbid conditions, participants self-reported all prescription and non-prescription medications, if medications were used to control conditions (i.e., high blood pressure, cholesterol, thyroid disorder), smoking behaviors, and alcohol use.

### Statistical Analyses

Participants were characterized by frailty status (i.e., non-frail, prefrail, frail). Chi-square tests were used to evaluate baseline characteristics, frailty status, and comorbidities. Associations between baseline characteristics and 1) being prefrail or frail or 2) having any comorbidity were examined in logistic regression models. Modified Poisson regression models with robust error estimates were used to estimate prevalence rates (PR) and 95% confidence intervals (CIs) for the independent associations between participant characteristics and frailty status, and between participant characteristics and comorbidities.[25] Demographic characteristics included in the models were age, sex, race/ethnicity, marital status, education, employment status, and the presence of a child in the home. Disease and medical characteristics included cancer type, cancer stage, time since diagnosis, and treatment modalities (i.e., surgery, chemotherapy, and radiation) along with self-reported comorbidities and medications. Lifestyle characteristics included smoking status, alcohol consumption, body mass index, and self-reported physical activity levels. All analyses were performed using SAS statistical software (V9.4, Cary, NC).

## RESULTS

### Participants

The majority of participants were non-Hispanic white women (82%) and between 35–39 years old (average 33.4±4.8) (Table 1). On average, participants were 3.7 (±2.4) years post-diagnosis. The most frequently reported types of cancer were solid tumors (67%) consisting of primarily breast, gynecologic, and thyroid, followed by leukemias and lymphomas (23%), and melanomas. Almost three-fourths (74%) of participants reported more than one type of treatment modality with surgery being the most prevalent, followed by chemotherapy, then radiation.

### Frailty

The prevalence of the frailty phenotype (≥ 3 components) was 14% among all participants and prefrailty (2 components) was present among 24% of participants (Table 1). Among all participants, the most frequently reported component of the frailty phenotype was fatigue (70%), followed by resistance (38%), ambulation (14%), and weight loss (13%). Only 2 participants met the criteria for illness (≥ 5 comorbidities). Prevalence of frailty components were similar across men and women with the exception of higher incidence of fatigue among women (73% vs. 59%, p = 0.05).

### Demographic and Lifestyle Characteristics Associated with Prefrailty and Frailty

The prevalence of prefrailty and frailty did not differ between men and women (25% prefrail and 10% frail vs. 23% prefrail and 14% frail, respectively), or by other sociodemographic factors. (Table 2). Participants with a BMI of 25–30 or BMI > 30 had an increased likelihood of prefrailty or frailty compared with individuals with BMI < 25 (BMI 25–30, PR: 1.65, 95% CI: 1.02–2.65; BMI > 30, PR: 2.36, 95% CI: 1.46–3.81). Compared to individuals reporting 0 minutes of MVPA, those who reported between 1–50 minutes/week (50th percentile) or over 50 weekly minutes of MVPA had lower likelihoods of being classified as prefrail or frail (PR: 0.62, 95% CI 0.43–0.90, and PR: 0.66, 0.42–1.03, respectively).

### Cancer Related Characteristics Associated with Prefrailty and Frailty

Cancer and treatment-related characteristics associated with frailty status included time since diagnosis and having more than one malignancy (Table 2). Being diagnosed with more than one malignancy was associated with over 2 times the likelihood of having prefrailty or frailty (PR: 2.31, 95% CI: 1.42–3.78).

### Comorbid Conditions Associated with Prefrailty and Frailty

Among the 104 IMPACT participants with prefrailty or frailty, 66% had at least one comorbidity (Table 3). None of the comorbidities examined in this sample were associated with frailty risk. However, having more than one comorbidity requiring medication was associated with an increased likelihood of having prefrailty or frailty (PR: 1.53, 95% CI 1.09–2.15). The most frequently reported comorbid condition was depression (38%), followed by a thyroid condition (19%), and hypertension (10%).

Having any comorbidity was associated with sex, relationship status, cancer type, having more than one malignancy, treatment modality, BMI, and smoking status (Table 4). Men were less likely than women to have reported at least one comorbid condition (59% vs. 37%) (PR: 0.63, 95% CI: 0.42–0.93). Participants that were single, widowed, separated, or divorced had increased likelihood of comorbidities compared to those that were partnered (PR: 1.27, 95% CI 1.01–1.60). Compared to other cancer types (i.e., leukemia/lymphomas, melanoma), individuals with solid tumors were more likely to have a comorbid condition (PR: 1.75, 95% CI: 1.03–2.98). Having more than one malignancy was associated with increased likelihood of reporting any comorbidity (PR: 1.72, 95% CI 1.28–2.31). Having a BMI of 30 or greater was associated with increased likelihood of comorbidities (PR: 1.31, 95% CI: 1.00–1.73) compared to BMI < 25. Being a current or former smoker was also associated with increased likelihood of reporting any comorbidity (PR: 1.29, 95% CI: 1.03–2.98).

## DISCUSSION

The frailty phenotype is an important prognostic tool to identify individuals at risk for adverse health outcomes and is an independent predictor of premature mortality in older populations.[6], [7] To our knowledge, our study is the first to evaluate the prevalence of frailty and prefrailty among a large cohort of YACS enrolled in a physical activity intervention trial. In this cross-sectional analysis of participants’ baseline data, we observed high prevalence of both frailty (14%), prefrailty (24%) and comorbid conditions (55%).

The overall prevalence of frailty and prefrailty in this sample of 280 YACS are consistent with the findings of Smitherman et al. in a cohort of AYA cancer survivors (10% frailty and 21% prefrail)[10] and among young adult survivors of childhood cancers (8% frail, 18–22% prefrail).[9] These prevalence estimates of frailty in the current study are higher than those found among older adults (11%) without a history of cancer.[9], [26] Systematic reviews of older adults from high-income countries, have found global frailty rates of 7–10%.[27][7] Across cohorts, frailty is higher among women, increases with age, and increases with the number and severity of comorbidities.[28]

Our findings concerning comorbidities in this population expand on prior work by Smitherman et al. that similarly found that prevalence was higher among women, and that women reported more medication use for comorbidity management.[10] The prevalence of at least one comorbidity was 55% in the present study, similar to the UNC AYA cohort (60%)[10] and lower than the St. Jude CCS cohort (82%).[9] Comorbidities may be higher in the St. Jude cohort due to diagnosis at earlier ages, longer times since diagnosis/age at assessment, and because individuals were specifically recruited to examine treatment-related cardiac and cognitive outcomes.[9] Additionally, we found similar prevalence of comorbidities requiring medication (39%) in our study as in the UNC AYA cohort (38%), indicating a need to identify and manage these conditions to prevent progression, chronic illness, and frailty.[10]

In this sample, self-reported BMI and MVPA were both associated with frailty status. Compared to participants with BMI < 25, those at every higher level of BMI had an increased likelihood of frailty status, ranging from a 1.7–2.4-fold increase. This finding is similar to previous studies showing frailty status was associated with having overweight and obesity for young adult cancer survivors and older adults without a history of cancer.[10], [29] We found that MVPA was associated with lower likelihood of being classified as frail or prefrail. This finding builds on prior work in this population that did not examine physical activity levels. Previous studies have demonstrated that the underlying physical symptoms of the frailty phenotype can be rehabilitated through physical activity and nutritional intervention.[30], [31] For instance, in older frail populations, physical activity programs and nutritional supplements have been shown to be effective for preventing the progression of frailty, improving frailty status, functional status, and mobility.[32] Our findings indicate that even in the absence of in-person or objective measures, self-reported physical activity levels and BMI can identify individuals at risk for comorbidities and frailty who might benefit from intervention. The high prevalence of frailty at baseline in a sample of YACS motivated to participate in a lifestyle intervention trial highlights the need to develop interventions to address frailty and mitigate adverse health outcomes in this population. Future work should prospectively examine whether engagement in PA over time is associated with reduced risk of frailty and prefrailty.

Several cancer-related characteristics emerged that could be useful in risk stratification in this population. Being diagnosed with more than one malignancy was associated with increased likelihood of comorbid conditions and frailty status. Further, having more than one comorbidity requiring medication management was associated with increased frailty. Compared to those 5 years post-diagnosis, corresponding to important clinical milestones for survivors, individuals were more likely to have frailty the closer they were to diagnosis. Prospective assessment of frailty early in survivorship could potentially be used for risk stratification, and to identify individuals in need of targeted interventions.

This is one of the few studies to evaluate frailty in YACS. A strength of this study was use of remote assessments, which enabled the recruitment of participants from around the United States and evaluation of a subgroup of cancer survivors that are understudied relative to other age groups. Additionally, the components used to evaluate the FRAIL index were consistent with previous versions, using measures that have been validated in the YACS population. This survey-based measure facilitated assessment of frailty status in a relatively large sample of YACS without requiring in-person assessments. In this cross-sectional study we characterized the prevalence of frailty status and comorbidities in a sample of YACS. However, the temporality of the relationships between participant characteristics, frailty status, and comorbidities cannot be determined with this analysis and should be the focus of future work.

Our findings should be interpreted with consideration of certain limitations. First, while our criteria for frailty is consistent with the FRAIL measure previously described [10], [33] (i.e., ≥ 5% weight loss in last 6 months), our time period for weight loss is shorter and potentially more conservative than other frailty classifications that consider unintentional weight loss of at least 10 pounds in the previous year.[6] In the current study, it was not possible to determine if weight loss in the previous six months was intentional or an indicator of low lean body mass as considered in other frailty measures including those used in the CCSC.[34], [35] Future validation of self-reported weight and objectively measured body composition is necessary to confirm reliability of this self-reported tool and establish relevant lean body mass cut points for this specific population. In general, there is a need for consensus around frailty measures and assessments.

Second, an eligibility criterion for the larger randomized controlled trial was not meeting the American Cancer Society’s recommendation of ≥ 150 minutes/week of MVPA for cancer survivors. Therefore, by nature of inclusion in the intervention, participants had to have low energy expenditure, which is a criterion for frailty in other measures. Similarly, our study sample volunteered to participate in an mHealth physical activity intervention trial and may be systematically different from YACS that would not choose to participate; they did not have comorbid conditions that would preclude them from participation, and they may be more active than some YACS while still being below guideline levels.

Data on comorbidities were collected for the purpose of screening for eligibility to the parent trial, and only two participants reported ≥ 5 comorbidities). The list of comorbidities assessed was not exhaustive, thus, there was potential for under identification of comorbidities as a component of the FRAIL index. Future research should assess non-cancer comorbidities more comprehensively among YACS and test validity and reliability of frailty measurement in this population. Ness et al. found that frailty was associated with the onset of new chronic conditions over time in the CCS cohort.[9] Identifying individuals with risk factors for frailty and providing appropriately tailored interventions could potentially prevent the onset or progression of chronic diseases, declines in physical function, and premature frailty.

In conclusion, we found that YACS enrolled in an mHealth physical activity intervention trial had high prevalence of frailty, prefrailty, and non-cancer comorbid conditions, similar to those found previously in samples of childhood cancer survivors and AYA cancer survivors.[10], [35], [36] These findings suggest the FRAIL index may be a useful tool for identifying individuals at risk for chronic diseases and morbidity. Results from this study indicate that among YACS, a higher likelihood of frailty was associated with increased BMI while engagement in any MVPA was associated with decreased likelihood of frailty. Findings may guide the development of future lifestyle interventions to address frailty in YACS by promoting improved body composition or physical activity.

## Figures and Tables

**Table 1:** Prevalence of Frailty and Prefrailty Among 280 Young Adult Survivors Participating in the IMPACT Trial

Frailty Component	Total (n = 280)		Non-frail, n = 176 (63%)		Prefrail, n = 66 (24%)		Frail, n = 38 (14%)	
	n	%	n	%	n	%	n	%
Fatigue	**197**	70	**101**	57	**58**	88	**38**	100
Resistance	**105**	38	**15**	9	**52**	79	**38**	100
Ambulation	**39**	14	**2**	1	**5**	8	**32**	84
Comorbidities	**2**	1	**0**	0	**1**	2	**1**	2
Weight Loss	**37**	13	**6**	3	**16**	24	**15**	39

**Table 2:** Associations between Demographic Characteristics and Comorbidities and Frailty Status Among 280 Young Adult Survivors Participating in the IMPACT Trial

Baseline Characteristic	Total n = 280	Non-frail n = 176 (63%)	Prefrail n = 66 (24%)	Frail n = 38 (14%)	*χ* ^2^	Prevalence of Frailty Status by Characteristics	
	*n (%)*	*n (%)*	*n (%)*	*n (%)*	*p*	*PR (95% CI)*	*p*
**Age at survey**					0.39		
18–29	**68** (24)	**45** (26)	**16** (24)	**7** (18)		0.76 (0.47–1.22)	0.25
30–34	**92** (33)	**60** (34)	**23** (35)	**9** (24)		0.78 (0.55–1.10)	0.15
35–39	**120** (41)	**71** (40)	**27** (41)	**22** (58)		*Ref.*	
**Sex**					0.67		
Female	**229** (82)	**143** (81)	**53** (80)	**33** (87)		*Ref.*	
Male	**51** (18)	**33** (19)	**13** (20)	**5** (13)		1.04 (0.65–1.64)	0.88
**Race/Ethnicity**					0.23		
Non-Hispanic White	**215** (77)	**141** (80)	**47** (71)	**27** (71)		*Ref.*	
BIPOC^a^	**65** (23)	**35** (20)	**19** (29)	**11** (29)		1.20 (0.79–1.82)	0.39
**Marital Status**					0.46		
Partnered	**173** (62)	**111** (63)	**42** (64)	**20** (53)		*Ref.*	
Single	**107** (38)	**65** (37)	**24** (36)	**18** (47)		1.10 (0.77–1.58)	0.59
**Education**					< 0.001		
< High School	**80** (29)	**39** (22)	**21** (32)	**20** (53)		1.11 (0.78–1.58)	0.56
College Degree	**101** (36)	**67** (38)	**22 (**33)	**12 (**32)		*Ref.*	
Graduate Degree	**99** (35)	**70 (**40)	**23 (**35)	**6 (**16)		0.79 (0.54–1.15)	0.22
**Occupation**					0.15		
Working	**192** (69)	**124** (70)	**47 (**71)	**21** (55)		*Ref.*	
Student	**35** (13)	**23** (13)	**8** (12)	**4** (11)		1.11 (0.65–1.89)	0.70
Not Working	**53** (19)	**29** (16)	**11** (17)	**13** (34)		1.05 (0.73–1.50)	0.80
**Household Income**					0.69		
< $30k	**45** (16)	**27** (15)	**8** (12)	**10** (26)		0.78 (0.48–1.27)	0.33
$30k – < $60k	**70** (25)	**44** (25)	**18** (27)	**8** (21)		0.76 (0.52–1.10)	0.15
≥ $60k	**147** (53)	**93** (53)	**36** (55)	**18** (47)		*Ref.*	
Prefer not to answer	**18** (6)	**12** (7)	**4** (6)	**2** (5)		0.83 (0.40–1.74)	0.63
**Health Insurance**					0.45		
Yes	**260** (93)	**166** (94)	**60** (91)	**34** (89)		*Ref.*	
No	**20** (7)	**10** (6)	**6** (9)	**4** (11)		0.78 (0.45–1.37)	0.39
**BMI**					< 0.001		
< 25	**89** (32)	**70** (40)	**14** (21)	**5** (13)		*Ref.*	
≥ 25 – < 30	**82** (29)	**53** (30)	**18** (27)	**11** (29)		1.65 (1.02–2.65)	0.04
≥ 30	**109** (39)	**53** (30)	**34** (52)	**22** (58)		2.36 (1.46–3.81)	0.01
**MVPA**					0.01		
0	**121** (43)	**63** (36)	**35** (53)	**23** (61)		*Ref.*	
1 – < 50 min/wk	**87** (31)	**61** (35)	**15** (23)	**11** (29)		0.63 (0.44–0.91)	0.01
≥ 50 min/wk	**72** (26)	**52** (30)	**16** (24)	**4** (11)		0.69 (0.45–1.06)	0.09
**Former/Current Smoker**					0.68		
Yes	**57** (20)	**33** (19)	**15** (23)	**9** (24)		0.91 (0.64–1.29)	0.58
No	**223** (80)	**143** (81)	**51** (77)	**29** (76)		*Ref.*	
**Consumes Alcohol**					0.14		
Yes	**186** (66)	**122** (69)	**44** (67)	**20** (53)		*Ref.*	
No	**94** (34)	**54** (31)	**22** (33)	**18** (47)		1.10 (0.80–1.53	0.55
**Cancer Type**					0.05		
Solid Tumors	**188** (67)	**121** (69)	**44** (67)	**23** (61)		1.12 (0.52–2.42)	0.77
Leukemia/lymphomas	**65** (23)	**33** (19)	**18** (27)	**14** (37)		1.46 (0.64–3.32)	0.36
Melanoma	**27** (10)	**22** (13)	**4** (6)	**1** (3)		*Ref.*	
**>1 Malignancy**					0.14		
Yes	**16** (6)	**7** (4)	**7** (11)	**2** (5)		2.31 (1.42–3.78)	< 0.01
No	**264** (94)	**169** (96)	**59** (89)	**36** (95)		*Ref.*	
**Stage at Diagnosis**					0.17		
I	**60** (21)	**42** (24)	**9** (14)	**9** (24)		*Ref.*	
II	**77** (28)	**42** (24)	**25** (38)	**10** (26)		1.39 (0.90–2.15)	0.14
III	**48** (17)	**36** (21)	**7** (11)	**5** (13)		0.71 (0.39–1.27)	0.24
IV	**27** (10)	**13** (7)	**9** (14)	**5** (13)		1.62 (0.92–2.86)	0.09
Other^b^	**68** (24)	**43** (23)	**16** (24)	**9** (24)		1.04 (0.65–1.66)	0.88
**Treatment Type**					0.04		
Surgery Only	**56** (20)	**43** (24)	**7** (11)	**6** (16)		*Ref.*	
Systemic Treatment^c^	**224** (80)	**133** (76)	**59** (89)	**32** (84)		1.57 (0.92–2.57)	0.10
**>2 Treatment Modality**					0.30		
≤2	**184** (66)	**120** (68)	**39** (59)	**25** (66)		*Ref.*	
3–5	**96** (34)	**56** (32)	**27** (41)	**13** (34)		1.10 (0.80–1.51)	0.56
**Time Since Diagnosis**					0.08		
< 1.6 years	**68** (24)	**44** (25)	**19** (29)	**5** (13)		1.38 (0.85–2.23)	0.19
1.6–3.2 years	**71** (25)	**41** (23)	**18** (27)	**12** (32)		1.92 (1.21–3.03)	0.01
3.2–5.15 years	**71** (25)	**42** (24)	**13** (20)	**16** (42)		1.72 (1.08–2.72)	0.02
> 5.15 years	**70** (25)	**49** (28)	**16** (24)	**5** (13)		*Ref.*	
**Comorbid Conditions**							
**Depression** *					0.01		
Yes	**107** (38)	**57** (32)	**28** (42)	**22** (58)			
No	**173** (62)	**119** (68)	**38** (58)	**16** (42)			
**Hypertension** *					0.02		
Yes	**27** (10)	**11** (6)	**10** (15)	**6** (16)			
No	**253** (90)	**165** (94)	**56** (85)	**32** (84)			
**Thyroid Condition** *					0.16		
**High Cholesterol** *					0.18		
**Type I/II Diabetes Mellitus** *					0.68		
**Heart attack/Stroke** *					0.08		
≥1 Comorbidity* (range 0–5)					0.01		
Yes	**155** (55)	**86** (49)	**40** (61)	**29** (76)			
No	**125** (45)	**90** (51)	**26** (39)	**9** (24)			
≥1 Medication* (range 0–5)					0.06		
Yes	**123** (44)	**68** (39)	**34** (52)	**21** (55)			
No	**157** (56)	**108** (61)	**32** (48)	**17** (45)			
≥1 Comorbidity Requiring Medication* (range 0–5)					0.02		
Yes	**110** (39)	**58** (33)	**32** (48)	**20** (53)			
No	**170** (61)	**118** (67)	**34** (52)	**18** (47)			

a BIPOC: Black, Indigenous, Asian, American Indian or Alaska Native, Other, or Multiple Races

b Other stage at diagnosis: I don’t know, Not staged, and Other

c Systemic treatment: chemotherapy, radiation, hormone therapy, other: immunotherapy, radioactive iodine, vaccine, medication)

* Percentages omitted from table due to data privacy concerns relating to small cell sizes. Items not included in multivariable models.

**Table 3:** Associations between Demographic, Lifestyle, and Cancer Characteristics and Having Any Comorbid Condition Among 280 Young Adult Survivors Participating in the IMPACT Trial

Baseline Characteristic	Total n = 280	Any Comorbidity, n = 155 (45%)	No Comorbidities, n = 125 (55%)	*χ* ^2^	Prevalence of Any Comorbidity by Characteristics	
	*n (%)*	*n (%)*	*n (%)*	*p*	*PR (95% C)*	*p*
**Age at survey**				0.37		
18–29	**68** (24)	**33 (**21)	**35** (28)		0.89 (0.65–1.23)	0.49
30–34	**92** (33)	**51** (33)	**41** (33)		1.02 (0.79–1.31)	0.89
35–39	**120** (43)	**71** (46)	**49** (39)		*Ref.*	
**Sex**				0.01		
Female	**229** (82)	**136** (88)	**93** (74)		*Ref.*	
Male	**51** (18)	**19** (12)	**32** (26)		0.63 (0.42–0.93)	0.02
**Race**				0.77		
Non-Hispanic White	**215** (77)	**118** (76)	**97** (78)		*Ref.*	
BIPOC^a^	**65** (23)	**37** (24)	**28** (22)		0.95 (0.73–1.24)	0.72
**Marital Status**				0.15		
Partnered	**173** (62)	**90** (58)	**83** (66)		*Ref.*	
Single	**107** (38)	**65** (42)	**42** (34)		1.27 (1.01–1.60)	0.04
**Education**				0.76		
< High School	**80** (29)	**47** (30)	**33** (26)		1.04 (0.78–1.37)	0.81
College Degree	**101** (36)	**54** (35)	**47** (38)		*Ref.*	
Graduate Degree	**99** (35)	**54** (35)	**45** (36)		1.02 (0.79–1.32)	0.88
**Occupation**				0.35		
Working	**192** (69)	**103** (66)	**89** (71)		*Ref.*	
Student	**35** (13)	**18** (12)	**17** (14)		1.06 (0.75–1.48)	0.75
Not Working	**53** (19)	**34** (22)	**19** (15)		1.15 (0.89–1.48)	0.28
**Household Income**				0.87		
< $30k	**45** (16)	**26** (17)	**19** (15)		0.92 (0.65–1.30)	0.64
$30k – < $60k	**70** (25)	**41** (26)	**29** (23)		1.00 (0.76–1.30)	0.97
≥ $60k	**147** (53)	**78** (50)	**69** (55)		*Ref.*	
Prefer not to answer	**18** (6)	**10** (6)	**8** (6)		0.85 (0.55–1.31)	0.46
**Health Insurance**				0.97		
Yes	**260** (93)	**144** (93)	**116** (93)		*Ref.*	
No	**20** (7)	**11** (7)	**9** (7)		1.01 (0.64–1.61)	0.96
**BMI**				0.17		
< 25	**89** (32)	**45** (29)	**44** (35)		*Ref.*	
≥ 25 – < 30	**82** (29)	**42** (27)	**40** (32)		1.14 (0.86–1.52)	0.37
≥ 30	**109** (39)	**68** (44)	**41** (33)		1.31 (0.99–1.73)	0.06
**MVPA**				0.59		
0	**121** (43)	**71** (46)	**50** (40)		0.95 (0.71–1.28)	0.75
1 – < 50 min/wk	**87** (31)	**47** (30)	**40** (32)		0.92 (0.67–1.25)	0.58
≥ 50 min/wk	**72** (25)	**37** (24)	**35** (28)		*Ref.*	
**Former/Current Smoker**				0.03		
Yes	**57** (20)	**39** (25)	**18** (14)		1.29 (1.01–1.64)	0.04
No	**223** (80)	**116** (75)	**107** (86)		*Ref.*	
**Consumes Alcohol**				0.99		
Yes	**186** (66)	**103** (66)	**83** (66)		1.13 (0.89–1.44)	0.32
No	**94** (34)	**52** (34)	**42** (34)		*Ref.*	
**Cancer Type**				0.05		
Solid Tumors	**188** (67)	**110** (71)	**78** (62)		1.75 (1.03–2.98)	0.04
Leukemia/lymphomas	**65** (23)	**36** (23)	**29** (24)		1.60 (0.91–2.80)	0.10
Melanoma	**27** (10)	**9** (6)	**18** (15)		*Ref.*	
**Stage at Diagnosis**				0.95		
I	**60** (21)	**36** (23)	**24** (19)		*Ref.*	
II	**77** (28)	**41** (26)	**36** (29)		0.79 (0.58–1.07)	0.13
III	**48** (17)	**26** (17)	**22** (18)		0.87 (0.62–1.22)	0.42
IV	**27** (10)	**15** (10)	**12** (10)		0.95 (0.63–1.44)	0.81
Other^b^	**68** (24)	**37** (24)	**31** (25)		0.93 (0.68–1.26)	0.63
**> 1 Malignancy**				0.02		
Yes	**16** (6)	**13** (8)	**3** (2)		1.15 (0.90–1.47)	0.26
No	**264** (95)	**142** (92)	**122** 98		*Ref.*	
**Treatment Type**				0.37		
Surgery Only	**56** (20)	**28** (18)	**28** (22)		*Ref.*	
Systemic Treatment^c^	**224** (80)	**127** (82)	**97** (78)		1.00 (0.73–1.37)	1.00
**> 2 Treatment Modality**				0.08		
≤2	**184** (66)	**95** (61)	**89** (71)		*Ref.*	
3–5	**96** (34)	**60** (39)	**36** (29)		1.15 (0.90–1.47)	0.26
**Time Since Diagnosis**				0.70		
< 1.6 years	**68** (24)	**39** (25)	**29** (23)		1.03 (0.77–1.37)	0.86
1.6–3.2 years	**71** (25)	**35** (23)	**36** (29)		0.95 (0.68–1.31)	0.74
3.2–5.15 years	**71** (25)	**41** (26)	**30** (24)		1.13 (0.85–1.50)	0.40
> 5.15 years	**70** (25)	**40** (26)	**30** (24)		*Ref.*	

a BIPOC: Black, Indigenous, Asian, American Indian or Alaska Native, Other, or Multiple Races

b Other stage at diagnosis: I don’t know, Not staged, and Other

c Systemic treatment: chemotherapy, radiation, hormone therapy, other: immunotherapy, radioactive iodine, vaccine, medication
